# Highly catalytic nanoenzyme of covalent organic framework loaded starch- surface-enhanced Raman scattering/absorption bi-mode peptide as biosensor for ultratrace determination of cadmium

**DOI:** 10.3389/fnut.2022.1075296

**Published:** 2023-01-09

**Authors:** Jingjing Li, Yiyi Shu, Chongning Li, Zhiliang Jiang

**Affiliations:** ^1^School of Public Health, Guilin Medical University, Guilin, China; ^2^Guangxi Key Laboratory of Environmental Pollution Control Theory and Technology, Guilin, China

**Keywords:** Cd^2+^, peptides, COF loaded-SS catalysis, SERS, gold nanoparticle, indicator reaction

## Abstract

High affinity peptides (PTs) have been used in nanoanalysis, but there are no reports which combine PTs with a liquid crystal (LC) covalent organic framework (COF) supported soluble starch (SS) catalytic amplification system as a biosensor recognition element. In this study, a new, highly sensitive and selective bi-mode molecular biosensor has been developed for the determination of cadmium ion (Cd^2+^). Specifically, a highly catalytic and stable COF supported SS nanosol catalyst was fabricated such that a nanocatalytic indicator reaction system for HAuCl_4_-sodium formate was established based on surface-enhanced Raman scattering (SERS). The Au nanoparticles produced exhibited a surface plasmon resonance (SPR) absorption peak at 535 nm and a SERS peak at 1,615 cm^–1^. Combining the nanocatalytic amplification indicator system with the specific PTs reaction permitted a sensitive and selective SERS/absorption bi-mode platform to be developed for the determination of cadmium in rice. The linear range for SERS determination was 0.025–0.95 nmol/L and the detection limit (DL) was 0.012 nmol/L.

## 1. Introduction

Peptides (PTs) which are widely available and of relatively low cost have good biocompatibility and water solubility properties. They also have a high affinity for many metal ions and have become excellent receptors for use in methodologies which exploit molecular recognition chemistry. Among them, absorption, fluorescence and electrochemical methods are used frequently in chemical analysis (1, 2). Zhuang et al. (3) reported a peptide fluorescent probe TP-2 (TPE-TrP-Pro-GlN-His-Glu-NH_2_), which utilized an aggregation-induced emission effect and afforded high selectivity in the determination of Hg^2+^, the detection limit (DL) being 41 nM. Yu et al. (4) immobilized an antibody against C-peptide onto TRF microspheres in a directional manner, thus fully exposing the antigen binding site, to determine C-peptide in human serum with a DL of 0.005 ng/mL. Furthermore, DNA and metal ions can be also determined by using PT-based sensors (5-7). However, to the best of our knowledge there are no reports on the use of a PT sensor for the determination of Cadmium ion (Cd^2+^) based on quantitative surface-enhanced Raman scattering (SERS) analysis, whereby a covalent organic framework (COF) loaded soluble starch (SS) facilitates nanocatalytic amplification.

SERS is a versatile analytical technique in the biosciences and affords high sensitivity, rapid and non-destructive analysis and multiple measurement capability (8-10). Based on the competitive binding between Au nanoparticles and RKGSGRRLVKC (11 peptide) and CALNN (5 peptide), SERS has been used to determine heparin (11), the analytical range being 0.2–2.4 μg/mL with a DL of 0.042 μg/mL. Liang et al. (12) studied the combination of catalytic graphene oxide nanoribbons (GONR) and human chorionic gonadotropin (hCG) polypeptide as a novel biosensor and established a new GONR-catalyzed RRS/absorption method to determine hCG with a DL of 70 pg/mL.

The COFs are a class of crystalline porous organic polymers that use small organic monomers as building blocks that are connected by covalent bonds. The pore structures, the strong light absorption ability and the tunable structures have attracted much interest recently (13, 14). In fact, COFs have been used as a multifunctional platform for gas adsorption and separation, sensing, and proton conduction, energy storage and heterogeneous catalysis (15, 16). The synthesis of COFs can be carried out using a variety of procedures including those based on solvothermal, ionothermal, and microwave energy (17). Most of these procedures, however, require relatively hazardous conditions and relatively long COF package crystallization times. As attractive synthesis routes, mechanical chemical polishing and hydrothermal treatment at room temperature are facilitating the extensive application and preparation of the COF package (18) as evidenced by recent studies (19-21) in optics, chemistry and electronics. Also, SERS has been applied to the determination of metal ions, based on the use of COF materials (22, 23). To improve the performance of the COFs, the doping of metal nanoparticles into the COFs is simple and effective strategy. In this approach, the number of free electrons on the surface of the COFs is increased, thereby facilitating the transfer of redox electrons and promoting the intended reaction (24). Starch is a common natural polydextrose (25, 26). In natural starch, amylose, which is soluble, accounts for 20∼26% of the total content, the remainder consisting of amylopectin. Oyamada et al. reported (26) that three amylose alkyl carbamate esters, namely, amylose triester (n-butylcarbamate), amylose triester (ethyl carbamate), and amylose triester (hexyl carbamate) in tetrahydrofuran form different liquid crystal (LC) phases that exhibit lyotropic liquid crystallinity. However, the thermotropic LC properties are rarely reported for amylose. Amylose has been used as an end sealer for metal and bimetal nanoparticles. Balasurya et al. (27) developed a novel core-shell Fe@Ag-starch nanosphere phenylalanine conjugate, and determined trace levels (1.84 nmol/L) of Hg^2+^ by absorption (Abs) spectrophotometry. Sapyen et al. (28) used SS-capped AgNPs in combination with a colorimetric method to determine trace levels (48 μg/L) of Cr (VI). Though starch as a natural polymer is cheap and may be used in nanoanalysis, little attention has been given to the stabilization of starch nanoparticles in catalytic reactions.

Cadmium ion is a toxic heavy metal and may be removed from polluted and contaminated waters by ion exchange, electrolysis, chemical precipitation, bioremediation or adsorption (29). However, chemical precipitation would result in sedimentation, which could then cause secondary pollution (30). Ion-exchange and electrolysis methods, while more efficient, have much higher maintenance costs (31). Therefore, to establish a highly sensitive and selective method for the determination of trace levels of Cd^2+^ is of great significance. Traditional analytical methods for the determination of Cd^2+^ include electrochemical, colorimetric, and fluorescence methods (32). In comparison with the aforementioned methods, biosensors are favored because of their low cost, real-time monitoring capability and low equipment requirements. In this work, the LC properties of SS and COF supported SS (COF_*TpBD*–*SS*_) were studied by resonance Rayleigh scattering (RRS) and Abs spectrophotometry. The COF_*TpBD*–*SS*_ was prepared for the first time as a nanoparticle enzyme in the liquid phase in order to facilitate a catalytic Au nanoreaction. A novel PT biosensor with high sensitivity and selectivity was then constructed by combining the COF_*TpBD*–*SS*_ with the PT biometric element to exploit the novel catalytic effect. Thereafter, a bi-mode biosensor platform was established, which afforded not only a SERS and Abs measurement capability but also was simple and of low-cost.

## 2. Experimental

### 2.1. Reagents and instrumentation

TU-1901 type dual-beam UV-Vis spectrophotometer (Beijing Puxi General Instrument Co., Ltd., Beijing, China); DXR Raman spectrometer (Thermo Fisher Scientific Company, Massachusetts, USA); Ultrasonic cleaner SK3300B (Shanghai Kedao Ultrasonic Instrument Co., Ltd., Shanghai, China); SYZ-550 type quartz sub-boiling water distiller (Jiangsu Jingbo Instrument Factory, Jiangsu, China); DHG-9023A electric heating constant temperature blast drying oven (Shanghai Jinghong Experimental Equipment Co., Ltd., Shanghai, China); HH-1 electric heating constant temperature water bath (Shanghai Weicheng Instrument Co., Ltd., Shanghai, China); DF-101S-type heat-collecting constant temperature heating magnetic stirrer (Gongyi Yuhua Instrument Co., Ltd., Shanghai, China); High speed refrigerated centrifuge (GL-25MS, Shanghai Luxiangyi Centrifuge Instrument Co., Ltd., Shanghai, China).

Benzidine (BD, Jilin China Science and Technology Co., Ltd., Jilin, China); 2,5-dimethoxyterephaldehyde (DMTP, Shanghai Macklin Biochemical Co., Ltd., Shanghai, China); (SS, Xilong Science Co., Ltd., Santou, China); amylopectin (AP, Xilong Science Co., Ltd., Santou, China); dimethyl sulfoxide (DMSO, Xilong Chemical Co., Ltd., Santou, China); 2,4,6-trihydroxy-1,3,5-benzenetricarbaldehyde (Tp, Shanghai Macklin Biochemical Co., Ltd., Shanghai, China); acetone (AT, Xilong Science Co., Ltd., Santou, China); tetrahydrofuran (THF, Xilong Science Co., Ltd., Santou, China); Benzene-1,3,5-tricarbaldehyde (BTCA, Shanghai Macklin Biochemical Co., Ltd., Shanghai, China); 1,3,5-tris (4-aminophenyl)benzene (TAPB, Shanghai Macklin Biochemical Co., Ltd., Shanghai, China); 0.1% HAuCl_4_ (1.0 mg/mL, National Group Chemical Reagent Co., Ltd., Shanghai, China); Peptide CW-Cd^2+^ (PT) sequence:Csy-Pro-Pro-Cys-Trp-NH_2_ (Shanghai Apeptide Co., Ltd., Shanghai, China); 10^–5°^mol/L Victoria Blue B (VBB) and 1 mol/L NaCl were used. The reagents used were of analytical grade, and the experimental water was sub-boiling distilled water.

### 2.2. Preparation of COF materials

#### 2.2.1. Preparation of COF_TpBD_

In a clean stoppered glass bottle (50 × 30), 6.5 mg of Tp (3 mmol/L), 20 mL of C_2_H_5_OH, and 2.5 mL of 3 mol/L acetic acid were added in succession. Dissolution of the reagents was achieved by agitation in an ultrasonic bath for 30 min. 8.3 mg of BD (4.5 mmol/L) was then added to the reaction mixture which was in an agitated state, and ultrasonic energy was applied again for 30 min. The reaction mixture was then washed with AT (3 × 10 mL) and THF (3 × 10 mL) successively and centrifuged at high speed (10,000 r/min, 10 min) to obtain a brown solid. The final product was freeze-dried for 21 h to obtain the brown COF_TpBD_ in powdered form.

#### 2.2.2. Preparation of COF_TpBD–SS_

Ten milliliter of 6.75 mmol/L BD solution, 10 mL of 4.5 mmol/L Tp solution and 10 mL of 3 mmol/L SS (the optimal conditions after optimization) were added to a clean corked glass bottle (50 × 30) while undergoing agitation for 30 min. A red-brown solid was obtained. Next, successive washings of the product with AT (3 × 10 mL) and THF (3 × 10 mL) were performed, and following high-speed low temperature centrifugation (10,000 r/min, 10 min), a yellow-brown solid was obtained. The final product was freeze-dried for 24 h, resulting in the COF_TpBD–SS_ powder which was red-brown in color.

#### 2.2.3. Preparation of COF_TpBD–AP_

Ten milliliter of 6.75 mmol/L BD solution, 10 mL of 4.5 mmol/L Tp solution and 10 mL of 3 mmol/L AP (the optimal conditions after optimization) were added to a clean corked glass bottle (50 × 30) which was placed in an ultrasonic bath and agitated for 30 min. An orange-brown product was obtained. The product was washed with AT (3 × 10 mL) and THF (3 × 10 mL) in turn, and centrifuged at 10,000 r/min for 10 min to obtain an orange-brown solid. The final product was freeze-dried for 24 h to yield the COF_TpBD–AP_ powder which was yellow-brown in color.

#### 2.2.4. Preparation of COF_TB_

19.6 mg of TAPB, 20 mL of DMSO and 1 mL of 3 mol/L HAc were added successively to a clean corked glass bottle (50 × 30) and the mixture was dissolved with the aid of ultrasonics for 30 min. Then, 10 mg of BTCA was added with mild stirring at room temperature, and the stirring continued for 1 min to obtain a yellow gel. The gel was next washed successively with AT (3 × 10 mL) and then THF (3 × 10 mL), and a dark yellow solid was obtained after high-speed and low temperature centrifugation (10,000 r/min, 10 min). The final product was freeze-dried for 21 h to yield the COF_TB_ powder which was yellow in color.

#### 2.2.5. Preparation of COF_BT–SS_

Ten milliliter of 8.4 mmol/L TAPB solution, 10 mL of 8.4 mmol/L BTCA solution and 8 mL of 3 mmol/L SS solution were successively in a clean corked glass bottle (50 × 30) which was agitated in an ultrasonic bath for 30 min. A yellow product was obtained and washed successively with AT (3 × 10 mL) and THF (3 × 10 mL), and a yellow solid was obtained after high-speed low temperature centrifugation (10,000 r/min, 10 min). The final product was freeze dried for 24 h to yield the COF_BT–SS_ which was a yellow powder.

#### 2.2.6. Preparation of COF_DT_

8.7 mg of DMTP, 20 mL of BA solution and 2.5 ml of 3 mol/L HAc were added successively to a clean corked glass bottle (50 × 30) and dissolved with the aid of ultrasonic agitation for 30 min. Then 10.5 mg of TAPB was added during stirring, and the stirring was continued for 2 h. Then the product was placed into an oven at 60 product h. The product was washed successively with AT (3 × 10 mL) and anhydrous THF (3 × 10 mL). A brown solid product was obtained after high-speed low temperature centrifugation (10,000 r/min, 10 min). The final product was freeze-dried for 24 h to yield the brown COF_DT_ powder.

#### 2.2.7. Preparation of COF_DT–SS_

Ten milliliter of DMTP solution, 10 mL of TAPB solution, 2.5 mL of 3 mol/L acetic acid solution and 8.5 mL of 3 mmol/L SS solution (the optimal condition after optimization) were added to a clean corked glass bottle (50 × 30) during stirring for 2 h. The reaction mixture was then washed successively with AT (3 × 10 mL) and THF (3 × 10 mL), and a yellow-brown solid was obtained after high-speed low temperature centrifugation (10,000 r/min, 10 min). The final product was freeze-dried for 24 h to yield the COF_DT–SS_ which was a yellow-brown powder.

### 2.3. Experimental procedures

One hundred and seventy microliter of 0.1 mg/mL COF_TpBD–SS_, 120 μL of 100 nmol/L PT, and various concentrations of CdCl_2_ were added successively to a 5 mL calibrated tube, followed by 70 μL of 1 mg/mL HAuCl_4_. Then 60 μL of 0.1 mol/L HCl followed by 120 μL of 0.1 mol/L Fo were then added; the mixture was shaken well and the volume made up to 2 mL. Next the solution was heated in a water bath at 80°C for 15 min. After the ice water was cooled to room temperature, 100 μL of 10^–5^ mol/L VBB and 100 μL of 1 mol/L NaCl were added. Then, the Raman spectrometer was used to record the SERS spectrum. The SERS intensity (I) at 1615 cm^–1^ was measured as was the SERS signal I_0_ for the blank control solution (solution without Cd^2+^) to allow the difference signal (ΔI = I–I_0_) to be calculated. In the case of spectrophotometric analysis, VBB was omitted and the absorbance (A) at 535 nm was measured. The absorbance of the blank solution A_0_ (solution without Cd^2+^) was also recorded. The difference in the absorbance values, ΔA = A-A_0_, was then calculated.

### 2.4. Sample preparation

Samples of rice grown in Hunan, Hubei, Guangxi and Wuchang were purchased from local stores. The rice samples were dried in an oven at 120°C for 8 h and ground with an agate mortar. Samples of 1.0 g (accurate to 0.1 mg) were transferred to 50 mL conical flasks containing 10 mL of mixed acid (vol HN0_3_/vol HClO_4_: 4:1) and left overnight (about 15 h). The samples were digested on a hot plate until white smoke appeared. When the solutions turned colorless and clear or slightly yellow, the samples were removed and left to cool before adjusting the volume to 10 mL. Finally, 100 μL aliquots of the digests were collected after filtration using a 0.45 μm microporous membrane filter.

## 3. Results and discussion

### 3.1. Principle of SERS/Abs bi-mode analysis

**FIGURE 1:**
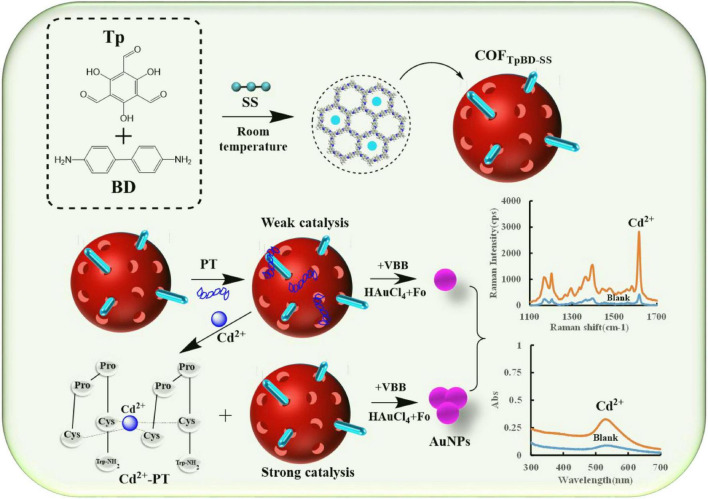
Schematic diagram of the SERS/Abs detection for Cd^2+^ coupled COF_TpBD–SS_ catalysis amplification *via* PT.

The COF material COF_TpBD_ was synthesized by stirring at room temperature using benzidine (Tp) and triuronic phloroglucinol (BD) as base reagents. Then SS was incorporated into the COF_TpBD_, and SS was uniformly loaded on the surface of COF_TpBD_ *via* hydrogen bond interaction to enhance the catalytic effect of COF_TpBD_. In general, in the absence of catalyst, Fo-HAuCl_4_ reacted slowly and its SERS signal was low. COF_TpBD–SS_ had a strong catalytic effect on the Fo-HAuCl_4_ reaction, and the AuNPs exhibited a prominent SERS effect. Moreover, PT could be adsorbed on the surface of COF_TpBD_, blocking contact between the active site and the reacting solution, thus inhibiting the catalytic effect. When Cd^2+^ was added to the solution, a stable conjugate was formed with PT, and COF_TpBD_ was released. The catalytic effect gradually recovered with increase of the Cd^2+^ concentration. The AuNPs generated gradually increased, and the AuNPs could be used as an active base for SERS. After adding the VBB probe molecules, the intensity of the SERS signal increased linearly as a function at 1,615 cm^–1^. Meanwhile, the absorbance at 535 nm was enhanced with an increase in the content of the AuNPs. Based on the SERS and the Abs responses, the possibility of developing a highly selective and bi-mode spectrochemical (SERS/Abs) method for the determination of trace levels Cd^2+^ was conceived (Figure 1).

### 3.2. Material characterization

#### 3.2.1. Electron microscopy

**FIGURE 2:**
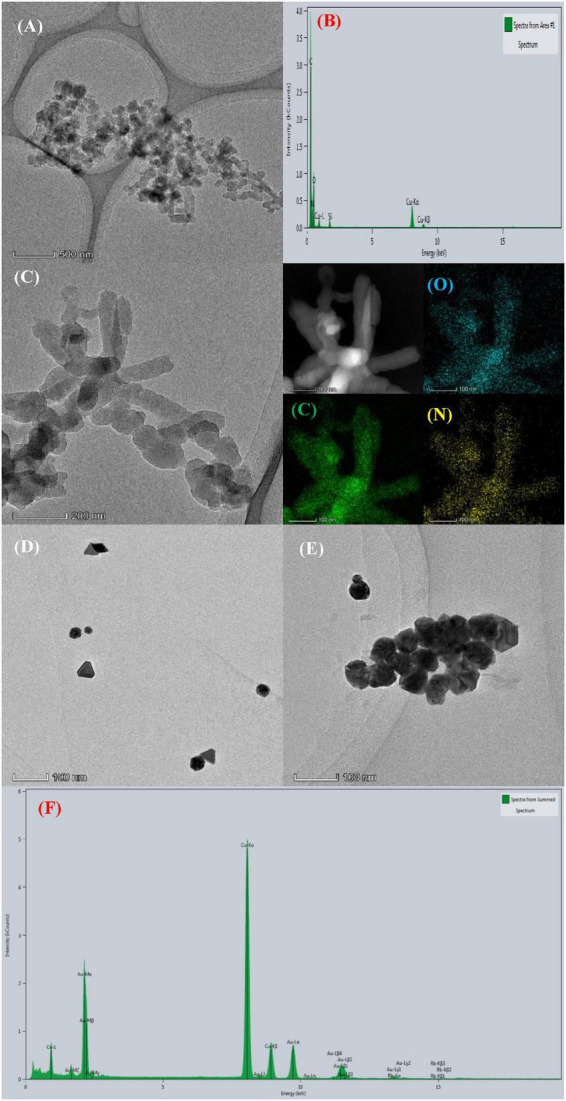
Transmission electron microscopy and energy dispersive X-ray analysis of the nanoparticles. **(A)** TEM of COF_TpBD_; **(B)** EDS of COF_TpBD_; **(C)** TEM of COF_TpBD–SS_; **(D)** TEM of analysis system: 5 nmol/L PT + 2.5 mmol/L HCl + 6.25 μg/mL COF_TpBD–SS_ + 0.025 mg/mL HAuCl_4_ + 5 mmol/L Fo; **(E)** TEM of analysis system: D + 0.045 nmol/L Cd^2+^; **(F)** EDS of analysis system.

The COF and the nano catalytic amplification system were analyzed by transmission electron microscopy (TEM) and energy dispersive X-ray analysis (EDS). As shown in Figure 2A, COF_TpBD_ has a rod and layered type structure with particles of size about 450 nm; also, the diffraction peaks corresponding to C, N, and O occurred at 0.26, 0.40, and 0.53 keV, respectively (Figure 2B). In addition, as shown in Figure 2C, the COF_TpBD_ doped SS did not change its morphology. Given that COF_TpBD–SS_ and COF_TpBD_ both contain elemental C, O, and N, it is not clear from the TEM and EDS spectra that SS is loaded on the surface of COF_TpBD_. However, it is clear that the SS is indeed loaded on the surface of COF_TpBD_ based on inspection of the spectra of the variable temperature molecular, Raman and infrared spectroscopies. The TEM images of the AuNPs generated by the reaction of the PT-COF_TpBD–SS_-AuNPs-Fo-HAuCl_4_ (Figures 2D, E) showed that in the absence of Cd^2+^, the AuNP were formed with an average particle size of 35 nm. In contrast, the addition of Cd^2+^ produced a large number of AuNPs particles with an average particle size of about 55 nm, as evidenced by the spectral peak at 2.2 keV (Figure 2F).

#### 3.2.2. Molecular spectra of nanomaterials

As shown in Supplementary Figures 1A, B, COF_TpBD_ had an absorption peak at 495 nm and SS had no absorption peak. After loading SS, the absorption peak of COF_TpBD–SS_ had a slight red shift and appeared at 500 nm relative to COF_TpBD_ (Supplementary Figure 1C). It can be seen from consideration of the above that the spectral results of the two molecules are consistent. The absorption peaks of AP and COF_TpBD–AP_ are shown in Supplementary Figures 1D, E. The UV variable temperature molecular absorption spectra of COF_TpBD_, SS and COF_TpBD–SS_ are presented in Supplementary Figures 1F–H. The Abs peak for COF_TpBD_ at 500 nm did not change with temperature, but the Abs peak for COF_TpBD–SS_ at 500 nm occurred within the temperature range 70–950e temperatwas added to COF_TpBD_. The absorbance increased with increase of temperature, but not before 70ea This indicated that COF_TpBD–SS_ exhibited the characteristics of a thermotropic LC (33), reflecting the fact that SS confers LC properties, and that some of SS molecules may be exposed on the COF surface. In Supplementary Figures 1I, J, the Abs peaks of AP and COF_TpBD–AP_ at 495 nm did not change with change in temperature indicating that AP and COF_TpBD–AP_ did not exhibit the characteristics of a thermotropic LC.

**FIGURE 3:**
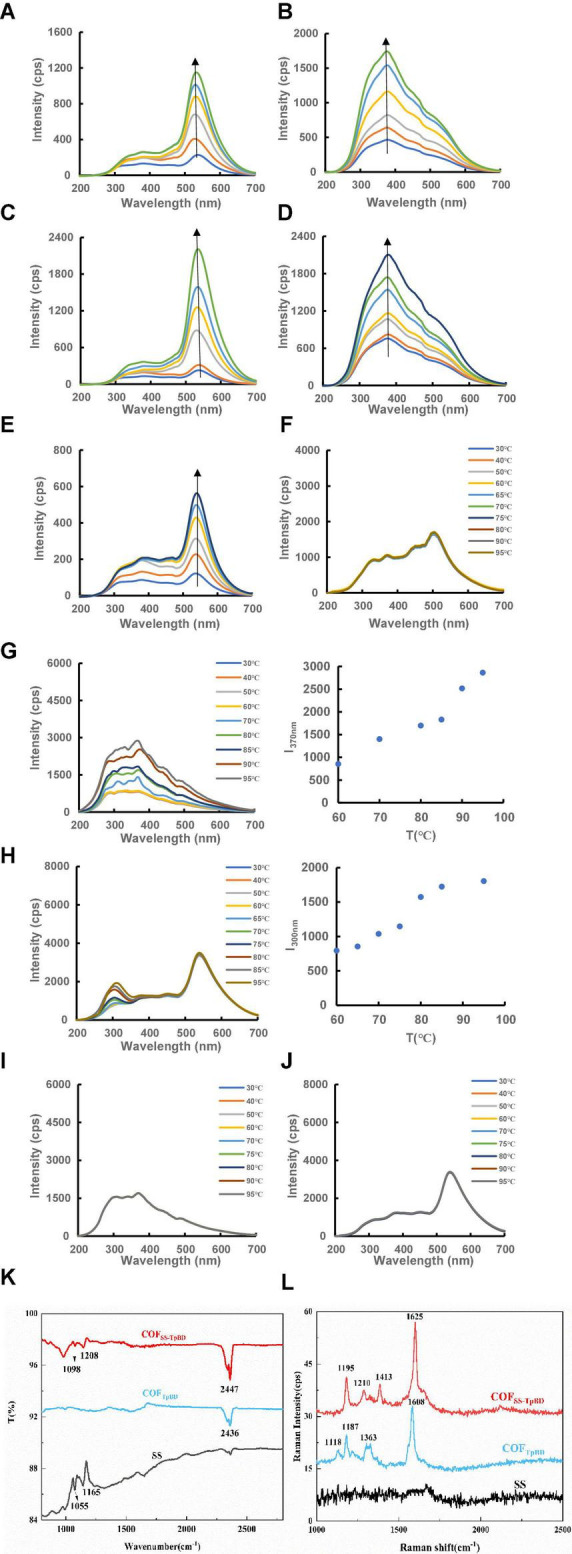
Resonance Rayleigh scattering spectra of COF_TpBD_/SS/COF_TpBD–SS_/AP/COF_TpBD–AP_ and FTIR and SERS spectra of OF_TpBD_/SS/COF_TpBD–SS_. **(A)** COF_TpBD_ RRS spectra, 0.35, 1.25, 6.25, 12.5, 20, 25 μg/mL; **(B)** SS RRS spectra, 0.35, 2.5, 4.25, 12.5, 20, 25 μg/mL; **(C)** COF_TpBD–SS_ RRS spectra 0.35, 0.7, 6.25, 12.5, 20, 25 μg/mL; **(D)** AP RRS spectra, 0.35, 1.25, 3.25, 6.25, 12.5, 20, 25 μg/mL; **(E)** COF_TpBD–AP_ RRS spectra, 0.35, 3.25, 6.25, 12.5, 20, 25 μg/mL; **(F)** The RRS signal for 30 μg/mL of COF_TpBD_ with variation in temperature; **(G)** The RRS signal for 30 μg/mL of SS with variation in temperature; **(H)** The RRS signal for 30 μg/mL of COF_TpBD–SS_ with variation in temperature; **(I)** The RRS signal for 30 μg/mL AP with variation in temperature; **(J)** The RRS signal for 30 μg/mL of COF_TpBD–AP_ with variation in temperature; **(K)** the FTIR of COF_TpBD_/SS/COF_TpBD–SS_; **(L)** the SERS signal for 10 μg/mL of COF_TpBD_/10 μg/mL SS/10 μg/mL COF_TpBD–SS_ with 0.1 mmol/L AuNP + 0.05 mol/L NaCl + 0.5 μmol/L VBB.

RRS is a simple and sensitive technique for studying nanoparticles such as LCs, hence was used in this work. COF_TpBD_ and SS generate a characteristic RRS peak at 535 and 370 nm (Figures 3A, B), respectively. By examining the RRS spectrum of COF_TpBD–SS_ (Figure 3C), it was found that after the loading of SS by COF_TpBD_, the characteristic peak of COF_TpBD_ was observed at 535 nm, while the RRS peak for SS at 370 nm was not clear. The variable temperature RRS spectra of COF_TpBD_ and SS are shown in Figures 3D, E, respectively. The RRS peak of COF_TpBD_ at 545 nm did not change with change of temperature, while the RRS peak of SS at 370 nm was unchanged before 60°C, but did change within the temperature range 60–95°C, the intensity of the RRS peak increasing with increase of temperature. This demonstrated that SS has LC properties. By investigating the variable temperature RRS spectra of COF_TpBD–SS_ (Figure 3F), it was found that after COF_TpBD_ was loaded with SS, two peaks appeared in the spectra, namely, at 300 and 535 nm. The two peaks did not change in appearance at temperatures less than 60°C. For temperatures between 60 and 95°C, the RRS intensity at 300 nm increased with increase in temperature (Figures 3G, H). The intensity of COF_TpBD–SS_ was weaker than that of the SS. However, the intensity of the COF_TpBD–SS_ peak at 535 nm remained constant. The results would suggest that COF_TpBD–SS_ had LC properties whereby some SS molecules were embedded into the COF porous structure, and some SS molecules may have been exposed on the COF surface. The variable temperature RRS spectra of AP and COF_TpBD–AP_ are presented in Figures 3I, J. The RRS peak of COF_TpBD–AP_ at 545 nm did not change with change of temperature, thus indicating that COF_TpBD–AP_ did not exhibit the characteristics of thermotropic LCs.

The infrared spectrum of COF_TpBD_/SS/COF_TpBD–SS_ is shown in Figure 3K. Due to the introduction of SS, the electronic structure of the surface underwent modification, hence the FTIR spectrum did change. As shown in Figure 3K, COF_TpBD_ exhibited characteristic infra-red peaks near 2,436 cm^–1^, which were attributed to the stretching vibration and in-plane rocking of the NH bond. The peaks for COF_TpBD–SS_ were at 1,098 and 1,208 cm^–1^ relative to COF_TpBD_. New peaks were formed, which were assigned to the C-O-C antisymmetric and symmetric stretching vibrations, indicating that the doping by SS may lead to changes in the surface structure of COF_TpBD_. The structure of COF was confirmed by the Raman spectra for the COF_TpBD_ and COF_TpBD–SS_ samples with AuNPs serving as the SERS substrate and NaCl as the aggregating agent (Figure 3L). COF_TpBD_ exhibited characteristic Raman peaks near 1,118, 1,187, 1,363, and 1,608 cm^–1^, which were attributed to the torsional vibration, the in-plane rocking of the CH bond, the stretching vibration of the benzene ring and the C = C stretching vibration, respectively. Characteristic Raman peaks for COF_TpBD–SS_ occurred at 1,195, 1,210, 1,413, and 1,625 cm^–1^, and the peaks belonged to the in-plane rocking of the CH bond, the stretching vibration of the C-O-C, the stretching vibration of the benzene ring and the C = C stretching vibration, respectively. The SS has no clear characteristic peaks in the Raman spectrum. Compared with COF_TpBD_, COF_TpBD–SS_ had a “new” characteristic peak at 1,210 cm^–1^, which was attributed to the stretching vibration of C-O-C. Due to the introduction of carbohydrate SS, the surface electronic structure had changed, hence the SERS spectrum changed. This indicated that a strong intermolecular force existed between the COF_TpBD_ and the SS, and the SS is well supported in the pores of COF_TpBD_; this factor may well account for the enhanced catalytic activity.

#### 3.2.3. Particle size distribution, surface charge analysis, and stability of COF_TpBD–SS_

The particle size distribution of COF_TpBD_ was measured using a particle size analyzer. The particle sizes of most COF_TpBD_ particles ranged from 300 to 800 nm, and the average size was 522 nm. The average particle size of COF_TpBD–SS_ was 1,412 nm after COF was loaded with SS, which reflects the incorporation of the long chain amylose (Supplementary Figure 2A). The charge distribution potentials for COF_TpBD_ and COF_TpBD–SS_ were 0.0652 and 0.11 mV, respectively (Supplementary Figures 2B, C). The larger the absolute potential became, the more stable were the particles in the solution, and hence the stability of COF_TpBD–SS_ was significantly improved (Supplementary Figure 2C).

To test the stability of COF_TpBD_, SS and COF_TpBD–SS_, changes in the charge distribution potential and their stability over time for solutions containing different concentrations of NaCl were studied, respectively. Supplementary Figure 2D shows that the absorbance values for COF_TpBD_ and SS and the mixed of SS and COF_TpBD_ solution decreased over time; the absorbance value for COF_TpBD–SS_ was, however, stable, and the change was less than 5%. To study the stability of these catalytic materials in NaCl solutions of different concentrations, 150 μL of 0.1 mg/mL COF_TpBD_, SS and COF_TpBD–SS_ were added into a series of 5 mL volumetric flasks. The absorbance signals were then measured by adding different volumes of 1 mol/L NaCl solution diluted with H_2_O to 2 mL (Supplementary Figure 2E). The COF_TpBD_ and SS samples were found to be unstable in the different concentrations of NaCl solution, while the absorbance value for COF_TpBD–SS_ was stable in low salinity solution. The absorbance value for the COF_TpBD–SS_ solution decreased slightly with increasing NaCl concentration. The results showed that COF_TpBD–SS_ was also stable in saline solution, while COF_TpBD_ and SS were relatively less stable. This was because SS could be evenly dispersed on the surface and in the pores of the COF_TpBD_ structure after COF_TpBD_ was loaded with SS, thus the stability of SS was greatly improved compared with that of COF_TpBD_ and SS.

#### 3.2.4. X-ray diffraction (XRD) and specific surface area of COF_TpBD_

Supplementary Figure 3A shows that COF_TpBD_ only has a diffraction peak near 25.7^°^, which corresponds to the (001) facet (34). Compared with COF_TpBD_, COF_TpBD–SS_ has no diffraction peaks of SS. This may be due to the low crystallinity of the loaded SS. To evaluate the permanent porosities and the surface area of COFs, the nitrogen adsorption-desorption isotherm was measured after degassing at 70°C for 6 h (Supplementary Figure 3B). According to the Brunauer–Emmett–Teller (BET) formula, the specific surface areas of COF_TpBD_ and COF_TpBD–SS_ are calculated to be 256 and 1,033 m^2^ g^–1^, respectively. The pore size distribution is illustrated in Supplementary Figure 3C, where the average pore sizes of COF_TpBD_ and COF_TpBD–SS_ are 7.9 and 3.2 nm, respectively. The existence of the mesoporous structure can easily expose active sites and facilitate full contact with the reactants, effectively improving the catalytic efficiency.

### 3.3. SERS/Abs spectra of nano catalytic amplification system

**FIGURE 4:**
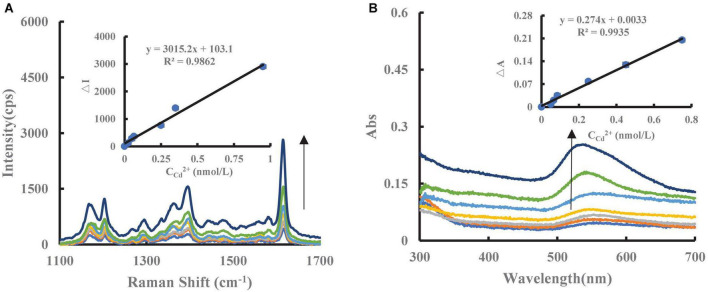
SERS/Abs spectra of catalytic amplification system. **(A)** (0, 0.025, 0.050, 0.065, 0.25, 0.35, 0.95) nmol/L Cd^2+^ + 5 nmol/L PT + 6.25 μg/mL COF_TpBD–SS_ + 2.5 mmol/L Fo + 0.5 mmol/L HCl + 0.025 mg/mL HAuCl_4_ + 0.5 μmol/L VBB + 0.05 mol/L NaCl; **(B)** Abs of (0, 0.05, 0.065, 0.085, 0.25, 0.45, 0.75) nmol/L Cd^2+^ + 5 nmol/L PT + 6.25 μg/mL COF_TpBD–SS_ + 5 mmol/L Fo + 2.5 mmol/L HCl + 0.025 mg/mL HAuCl_4_.

Under experimental conditions, the AuNPs generated by the Cd^2+^-PT-Fo-COF_TpBD_/SS/COF_TpBD–SS_/ COF_TB_/COF_TB–SS_/COF_DT_/COF_DT–SS_-HAuCl_4_ exhibited a strong SERS peak at 1,615 cm^–1^. After adding PT to the catalytic system, due to the electrostatic effect, the PT would be adsorbed on the surface of the catalyst, resulting in a decrease in the catalytic activity and a reduction in the number of AuNPs generated, hence a decrease in the SERS signal occurred. When the unknown sample (test solution containing trace level of Cd^2+^) was added to the inhibition system, PT was bound specifically to Cd^2+^ forming a stable structure, which detached from the catalyst surface. At the same time, the catalytic activity was restored, resulting in an increase in the number of the AuNPs generated by the nano system hence a recovery of the SERS signal (Figure 4A). Therefore, based on these SERS responses, it may be possible to establish a novel method for the measurement of Cd^2+^. In addition, the SERS spectra of the COF_TB_/COF_TB–SS_/COF_DT_/COF_DT–SS_- HAuCl_4_ system were recorded when different catalyst systems were used (Supplementary Figures 4A–F), however, as can be seen, the COF_TpBD–SS_ system exhibited the largest slope. For the experimental conditions used, a surface plasmon resonance (SPR) absorption peak of Cd^2+^-PT-COF_TpBD–SS_-Fo-HAuCl_4_ was observed at 535 nm (Figure 4B). This corresponded to the SPR absorption peak of the AuNPs.

### 3.4. Catalytic mechanism for COF_TpBD–SS_

Regarding COF_TpBD_, the particle size distribution observed was from 710 to 1990 nm for a solution of 6.25 μg/mL of COF_TpBD–SS_ at 25^°^C. For a temperature of 80^°^C, the size distribution was from 100 to 340 nm. The average particle size of COF_TpBD–SS_ was 286 nm. That is, an increase in temperature resulted in a smaller particle size, which correspondingly enhanced the catalytic activity of COF_TpBD–SS_. Therefore, in the case of large size particles, the nanocatalytic reaction did not readily take place at room temperature, and hence cooling of the solution may be used to suppress or stop the nanocatalytic reaction. For the SS, the plot for the change of size with temperature was similar to that for COF_TpBD–SS_ (Supplementary Figure 5).

Under the experimental conditions, the reaction of Fo and HAuCl_4_ is slow, in which Au^3+^ is reduced to Au (I) and Au, and HCOO^–^ is oxidized to CO_2_. COF_TpBD_/SS/COF_TpBD–SS_/COF_TB_/COF_TB–SS_/COF_DT_/COF_DT–SS_ catalyzes the HAuCl_4_-Fo reaction (Supplementary Table 1). However, the AP and COF_TpBD–AP_ did not exhibit catalytic activity. Regarding the slope of the linear equation in Supplementary Table 1, the slope of the COF_TpBD–SS_ catalytic system is greater than the slope for the sum of COF_TpBD_ and SS, thus demonstrating an enhanced catalytic effect.

**FIGURE 5:**
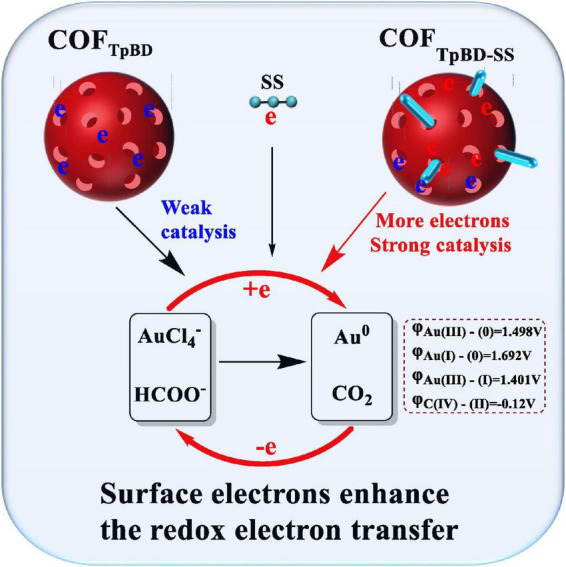
COF_TpBD–SS_ nanocatalytic enhancement mechanism.

A low concentration of a SS solution has a relatively small particle size, hence the surface electrons can enhance the redox electron transfer in the HAuCl_4_-Fo reaction (*cf* slope of 120.2). As is well known, SS is relatively unstable and can readily self-aggregate. When SS is loaded on the surface of COF_TpBD_, its stability and the catalytic effect are greatly improved, and the slope of COF_TpBD–SS_ is 303.9. Given that COF_TpBD_ has abundant pores and C = N bonds, there are a large number of π bonds and it is rich in π electrons, hence more active sites would be available for SS to be loaded on the surface, the size being limited by the pores. COF_TpBD_ acts as a carrier to make SS more evenly distributed and prevent its aggregation. When the particle size is at the nanometer scale, with increase in the number of surface atoms, the number of unsaturated bonds increase and the surface energy increases. The free electrons on the SS surface undergo coupling with the π electrons on the surface of COF_TpBD_. The two sources of electrons greatly facilitate the transfer of electrons in the redox reaction of the COF_TpBD–SS–_AuNPs, a process which is accelerated by the generation of AuNPs in the reaction hence the greatly enhanced catalytic activity (Figure 5). After PT is added to the nanosystem, due to the electrostatic effect, PT will be adsorbed on the surface of the catalyst and inhibit the catalytic activity. With increase in the PT concentration, the concentration of free catalyst in the system would decrease, and the catalytic activity would be weakened. Therefore, the AuNPs generated in the system would decrease as would the SERS signal (Supplementary Table 2), thus the inhibitory effect of PT was consistent with the observations of the catalytic effect. The main reactions are as follows:


(1) $$\mathrm{Au}\,\left(\mathrm{III}\right)+\mathrm{Fo}\,\underset{\to }{{\text{COF}}_{\mathrm{TpBD}-\mathrm{SS}}}\,A\,u\,\left(I\right)+{\text{CO}}_{2}$$

(2) $$\mathrm{Au}\,\left(I\right)+\mathrm{Fo}\,\underset{\to }{{\text{COF}}_{\mathrm{TpBD}-\mathrm{SS}}}\,A\,u+{\text{CO}}_{2}$$

(3) nAu=AuNPs

The relative standard electrode potential with common valence states of Au can be seen, E_0_ (Au^3+^/Au^0^) = 1.498 V; E_0_ (Au^+^/Au^0^) = 1.692 V; E_0_ (Au^3+^/Au^+^) = 1.401 V. Therefore, due to the low redox potential of trivalent gold ion (Au^3+^), it is difficult to reduce to gold nanoparticles (Au^0^) in one step. As shown in Supplementary Figure 6, under the same conditions, Au^3+^ and Au^+^ can be reduced by HCOO^–^ to form Au^0^. After the addition of COF_TpBD–SS_ nanocomposite as catalyst, due to its large specific surface area and more active site, it can promote the reaction faster, accelerate the reduction of Au^3+^ into Au^+^, and Au^+^ into Au^0^, and the reaction is easier to proceed.

### 3.5. Selection of conditions

#### 3.5.1. Preparation conditions for COF_TpBD–SS_

According to the experimental method, based on the strength of the catalytic effect of COF_TpBD–SS_ nanosol on the Au-nanoindicator reaction, the corresponding conditions which yielded a strong catalytic effect were selected. The preparation conditions for the catalyst were optimized by changing the experimental conditions, respectively. When the loading condition for COF_TpBD_ with SS was 10 mL of 3 mmol/L (Supplementary Figure 7A), the amount of SS in COF_TB_ was 8 mL of 3 mmol/L (Supplementary Figure 7B), and the amount of SS in COF_DT_ was 8.5 mL of 3 mmol/L (Supplementary Figure 7C), then the ΔI of the catalytic system was maximized Therefore, these conditions were selected as the optimum experimental conditions.

#### 3.5.2. Analysis conditions

The experimental conditions for SERS analysis were checked according to the experimental procedures. When the concentrations of the reactants/reagents were as follows: 6.25 μg/mL COF_TpBD–SS_ (Supplementary Figure 8A), 5 nmol/L PT (Supplementary Figure 8B), 0.025 mg/mL HAuCl_4_ (Supplementary Figure 8C), 2.5 mmol/L HCl (Supplementary Figure 8D), 5 mmol/L Fo (Supplementary Figure 8E), 0.5 μmol/L VBB (Supplementary Figure 8F), 0.05 mol/L NaCl (Supplementary Figure 8G), reaction temperature of 80°C Supplementary Figure 8H), reaction time of 15 min (Supplementary Figure 8I), the SERS response was optimal. The aforementioned conditions were chosen for SERS measurement.

### 3.6. Working (or analytical) curves

Using the optimized conditions, the corresponding ΔI/ΔA values for different concentrations of Cd^2+^ was plotted to obtain the working curve. It can be seen from Supplementary Tables 2, 3 that the slope of the working curve of the COF_TpBD–SS_-Cd^2+^ system was 1 times that of COF_TpBD_ and 3 times that of SS, respectively, indicating that the former was the most sensitive (Figure 4A). The linear equation for spectrophotometric measurement was ΔI_1,615_ *_cm_*^–1^ = 3015.2C + 103.1, and the linear range was 0.025–0.95 nmol/L. Although the reaction time for the spectrophotometric method was relatively short, the sensitivity was less than SERS by a factor (Supplementary Table 3). Compared with the reported molecular spectroscopic methods for the determination of Cd^2+^ (35-37), the present SERS method is highly sensitive method (Supplementary Table 4).

### 3.7. Reproducibility and specificity

Reproducibility is an important indicator to be considered by biosensors, and it is only feasible with small coefficient of variation (CV, also called relative standard deviation) (38). The intra-batch and inter-batch differences of SERS/Abs tests were conducted under 0.25 nmol/L cadmium standard solution (Supplementary Figure 9). In the same batch of tests, the Cd^2+^ was detected for 6 times. The results showed no difference, indicating that the intra-batch difference between individual biosensor tests was very small (Supplementary Figure 9A). In different 8 batches of tests, the Cd^2+^ was detected within the scope of effective detection (usually CV values < 15%) at 0.25 nmol/L cadmium standard solution, suggesting that the inter-batch difference was within the acceptable range (Supplementary Figure 9B). The potential for interferences by coexisting ions on the SERS determination of 0.01 nmol/L Cd^2+^ was investigated. The experimental results indicated that in the presence of: (1) 1,000-fold excess of the following ions Mg^2+^, Zn^2+^, K^+^, Al^3+^, Na^+^, Ca^2+^, Ba^2+^, Fe^3+^, Mn^2+^, Co^2+^, Cu^2+^, (2) 100-fold excess of Cr^6+^, NH_4_^+^, Hg^2+^, SO_4_^2–^, NO_2_^–^, I^–^, and (3) 10-fold excess of CO_3_^2–^, PO_4_^3–^, Br^–^, the aforementioned ions at the specified concentrations did not interfere with the measurement (Supplementary Table 5). Moreover, the relative error was within ± 10% hence the method afforded good selectivity for the determination of trace amounts of Cd^2+^.

### 3.8. Determination of cadmium in rice

After sample pretreatment, in order to perform a recovery test, aliquots of standard solutions of Cd^2+^ were added to the digests. The Cd^2+^ content was determined according to the 2.3 experimental procedures. The analytical results are shown in Supplementary Table 6. The Cd^2+^ contents of the digests ranged from 0 to 0.31 nmol/L, which corresponded to Cd^2+^ contents in rice of 0–12.21 ng/g. The relative standard deviations (RSDs) ranged from 1.9 to 9.7%, and the recoveries were 91.2–109.7%, confirming that the SERS assay was accurate and reliable.

## 4. Conclusion

Based on loading SS nanoparticles into COFs, a novel COF_TpBD–SS_ nanomaterial has been prepared. The nanomaterial not only enhanced catalytic activity but also had good stability. A nanoAu indicator reaction for COF_TpBD–SS_ catalysis was investigated by molecular spectroscopy such that a SERS/Abs bi-mode method was developed for the determination of Cd^2+^ in rice based on exploiting the highly sensitive nanocatalytic amplification reaction based on the highly selective reaction with PT. The SERS analysis method was sensitive, whereas the Abs was relatively simple and of low cost. A plausible nanocatalytic mechanism was proposed whereby the nanosurface electrons enhanced the redox electron transfer to speed up the AuNP reaction.

## Data availability statement

The original contributions presented in this study are included in the article/Supplementary material, further inquiries can be directed to the corresponding authors.

## Author contributions

JL and YS: software, visualization, writing—original draft, investigation, and formal analysis. CL and ZJ: software, visualization, conception, reviewing and editing, software, methodology, validation, formal analysis, and supervision. All authors contributed to the article and approved the submitted version.
