# Supplementary material for: Highly catalytic nanoenzyme of covalent organic framework loaded starch- surface-enhanced Raman scattering/absorption bi-mode peptide as biosensor for ultratrace determination of cadmium
Source: Front Nutr. 2023 Jan 9;9:1075296. doi: 10.3389/fnut.2022.1075296 (PMC9870315; doi:10.3389/fnut.2022.1075296)
Supplement: Supplementary file 1 [file Data_Sheet_1.docx]

**Highly catalysis nanoenzyme of COF loaded starch-SERS/Abs dimode peptide biosensor for ultratrace** **Cd^2+^**

**Jingjing Li^1,2^, Yiyi Shu^1,2^, Chongning Li^1,2*^, Zhiliang Jiang^1,2*^**

^1^ School of Public Health, Guilin Medical University, Guilin 541199, China; ^2^Guangxi Key Laboratory of Environmental Pollution Control Theory and Technology, Guilin 541006, China.

**a**

**f**

**a**

**g**

**a**

**f**

**a**

**f**

**a**

**f**

**(F)**

**(E)**

**(D)**

**(C)**

**(B)**

**(A)**

**(H)**

**(G)**

**(J)**

**(I)**

**FIGURE S1.** Abs spectra and temperature-varying Abs spectra of COF_TpBD_/SS/COF_TpBD-SS_/AP/COF_TpBD-AP_. **(A)** COF_TpBD_ Abs spectra, a-g: 0.35, 1.25, 3.25, 6.25, 12.5, 20, 25 µg/mL; **(B)** SS Abs spectra, a-f: 0.35, 3.25, 6.25, 12.5, 20, 25 µg/mL; **(C)** COF_TpBD-SS_ Abs spectra, a-f: 0.35, 3.25, 6.25, 12.5, 20, 25 µg/mL; **(D)** AP Abs spectra, a-f: 0.35, 1.25, 3.25, 6.25, 12.5, 25 µg/mL; **(E)** COF_TpBD-AP_ Abs spectra, a-f: 0.35, 3.25, 6.25, 12.5, 20, 25 µg/mL; **(F)** The Abs signal of 15 µg/mL COF_TpBD_ varying with temperature; **(G)** The Abs signal of 15 µg/mL SS varying with temperature; **(H)** The Abs signal of 15 µg/mL COF_TpBD-SS_ varying with temperature; **(I)** The Abs signal of 15 µg/mL AP varying with temperature; **(J)** The Abs signal of 15 µg/mL COF_TpBD-AP_ varying with temperature.


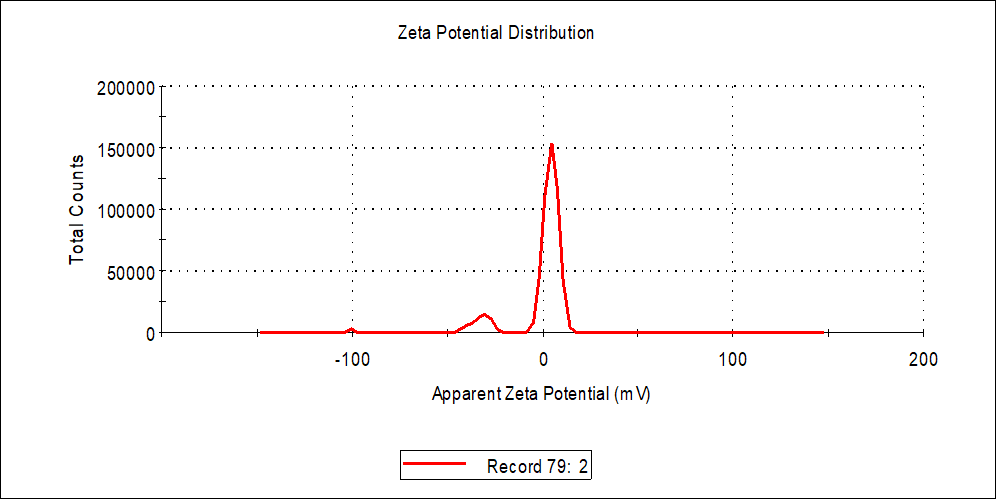

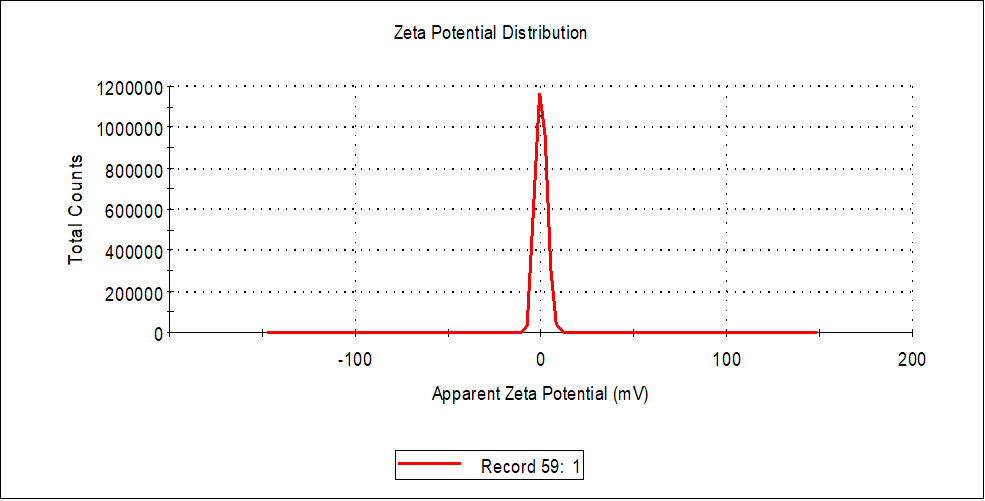


**(C)**

**(B)**

**(A)**

**(E)**

**(D)**

**FIGURE S2.** Particle size, surface charge analysis and stability of COF_TpBD_/SS/COF_TpBD-SS_ the mixture of COF_TpBD_ and SS. **(A)** Particle size analysis of COF_TpBD_ and COF_TpBD-SS_; **(B)** Zeta potential of COF_TpBD-SS_; **(C)** Zeta potential of COF_TpBD_; **(D)** The Abs signal of COF_TpBD_/SS/COF_TpBD-SS_/the mixture of SS and COF_TpBD_ varying with time; **(E)** The Abs signal of COF_TpBD_/SS/COF_TpBD-SS_/the mixture of SS and COF_TpBD_ varying with NaCl.

**(A)**

**(C)**

**(B)**

**FIGURE S3.** XRD, specific surface area and pore size distribution of COF_TpBD_/COF_TpBD-SS_. **(A)** XRD of COF_TpBD_ and COF_TpBD-SS_; **(B)** N_2_ adsorption–desorption isotherm of COF_TpBD_ and COF_TpBD-SS_; **(C)** pore size distribution of COF_TpBD_ and COF_TpBD-SS_.

**(B)**

**(A)**

**(D)**

**(F)**

**(E)**

**(C)**

**FIGURE S4.** SERS spectra of catalytic amplification system. **(A)** (0, 0.08, 0.09, 0.1, 0.35, 0.65, 0.85) nmol/L Cd^2+^ + 5 nmol/L PT + 6.25 µg/mL COF_TpBD_ + 5 mmol/L Fo + 2.5 mmol/L HCl + 0.025 mg/mL HAuCl_4_ + 0.5 µmol/L VBB + 0.05 mol/L NaCl; **(B)** (0, 0.085, 0.09, 0.15, 0.25, 0.55, 0.9) nmol/L Cd^2+^ + 5 nmol/L PT + 6.25 µg/mL COF_DT_ + 5 mmol/L Fo + 2.5 mmol/L HCl + 0.025 mg/mL HAuCl_4_ + 0.5 µmol/L VBB + 0.05 mol/L NaCl; **(C)** (0, 0.08, 0.09, 0.1, 0.2, 0.4, 0.9) nmol/L Cd^2+^ + 5 nmol/L PT + 6.25 µg/mL COF_TB_ + 5 mmol/L Fo + 2.5 mmol/L HCl + 0.025 mg/mL HAuCl_4_ + 0.5 µmol/L VBB + 0.05 mol/L NaCl; **(D)** (0, 0.025, 0.05, 0.065, 0.25, 0.35, 0.95) nmol/L Cd^2+^ + 5 nmol/L PT + 6.25 µg/mL COF_TpBD-SS_ + 5 mmol/L Fo + 2.5 mmol/L HCl + 0.025 mg/mL HAuCl_4_ + 0.5 µmol/L VBB + 0.05 mol/L NaCl; **(E)** (0, 0.055, 0.065, 0.085, 0.2, 0.35, 0.95) nmol/L Cd^2+^ + 5 nmol/L PT + 6.25 µg/mL COF_DT-SS_ + 5 mmol/L Fo + 2.5 mmol/L HCl + 0.025 mg/mL HAuCl_4_ + 0.5 µmol/L VBB + 0.05 mol/L NaCl; **(F)** (0, 0.035, 0.065, 0.1, 0.25, 0.35, 0.9) nmol/L Cd^2+^ + 5 nmol/L PT + 6.25 µg/mL COF_TB-SS_ + 5 mmol/L Fo + 2.5 mmol/L HCl + 0.025 mg/mL HAuCl_4_ + 0.5 µmol/L VBB + 0.05 mol/L NaCl.

**FIGURE S5.** Particle size distribution of 6.25 µg/mL SS/COF_TpBD-SS_ at 25 ^o^C and 80 ^o^C.

**FIGURE S6**. SERS spectrum. a: 0.025 mg/mL HAuCl_4_+2.5 mmol/L Fo; b: 0.025 mg/mL AuCl+2.5 mmol/L Fo; c: 0.025 mg/mL AuCl+2.5 mmol/L Fo+6.25 μg/mL COF_TpBD-SS_; d: 0.025 mg/mL HAuCl_4_+2.5 mmol/L Fo+0.04 μg/mL COF_TpBD-SS_.

**(C)**

**(B)**

**(A)**

**FIGURE S7.** Selection of the preparation condition. **(A)** the amount of SS load in COF_TpBD_; **(B)** the amount of SS load in COF_TB_; **(C)** amount of SS load in COF_DT_.

**(C)**

**(D)**

**(F)**

**(E)**

**(B)**

**(A)**

**(G)**

**(H)**

**(I)**

**FIGURE S8.** Effect of analytical comditions. **(A)** COF_TpBD-SS_; **(B)** PT; **(C)** HAuCl_4_; **(D)** HCl; **(E)** Fo; **(F)** VBB; **(G)** NaCl; **(H)** reaction temperature; **(I)** reaction time.

Coefficient of variation (CV) = (S.D./Mean)×100%, Mean: Average of different repetitions or different batches. S.D.: Standard deviation of different repetitions or different batches.

**(B)**

**(A)**

**FIGURE S9.** Reproducibility tests of SERS/Abs. **(A)** Intra-batch difference test, the same batch of devices with different repetitions; **(B)** Inter-batch differences, differences between different batches.

**TABLE S1. Comparison of catalytic characteristics of catalysts for HAuCl_4_-Fo reaction**

| **Catalysts** | **Linear equation** | **Linear range (µg/mL)** | **Coefficient (R^2^)** |
| --- | --- | --- | --- |
| COF_TpBD-SS_ | ΔI_1615cm_^-1^=303.9C- 158.6 | 1.5-9.5 | 0.9910 |
| COF_TpBD_ | ΔI_1615cm_^-1^=88.5C+ 18.2 | 1.35-9.25 | 0.9921 |
| SS | ΔI_1615cm_^-1^=120.2C- 49.9 | 2-10 | 0.9901 |
| AP | No catalyst | | |
| COF_TpBD-AP_ | No catalyst | | |
| COF_TB-SS_ | ΔI_1615cm_^-1^=180.3C- 89.0 | 1.25-9.75 | 0.9879 |
| COF_TB_ | ΔI_1615cm_^-1^=71.6C- 22.8 | 1.45-9 | 0.9893 |
| COF_DT-SS_ | ΔI_1615cm_^-1^=209.6C- 52.9 | 1.25-9.25 | 0.9883 |
| COF_DT_ | ΔI_1615cm_^-1^=76.5C- 20.2 | 1.25-9 | 0.9887 |

**TABLE S2. Comparison of aptamer inhibition characteristics**

| **Inhibition of system** | **Linear equation** | **Linear range（nmol/L）** | **Coefficient (R^2^)** |
| --- | --- | --- | --- |
| PT -COF_TpBD-SS_ -Fo | ΔI_1615cm_^-1^=225.8C -3.1 | 1-10 | 0.9930 |
| PT -COF_TpBD_ -Fo | ΔI_1615cm_^-1^= 80.3C+ 14.5 | 2.5-8.5 | 0.9887 |
| PT -SS-Fo | ΔI_1615cm_^-1^= 113.3C+ 65.5 | 1.5-9 | 0.9855 |
| PT -COF_TB-SS_ -Fo | ΔI_1615cm_^-1^= 167.3C+ 36.9 | 1-9.5 | 0.9835 |
| PT - COF_TB_ -Fo | ΔI_1615cm_^-1^= 73.3C+ 39.2 | 1.45-9.25 | 0.9863 |
| PT -COF_DT-SS_ -Fo | ΔI_1615cm_^-1^= 175.5C+ 79.9 | 1.25-9.5 | 0.9840 |
| PT -COF_DT_ -Fo | ΔI_1615cm_^-1^=76.1C+ 3.2 | 1.35-9.5 | 0.9873 |

**TABLE S3. Analysis characteristics of Cd^2+^ detection by SERS /Abs**

| **Catalytic system** | **Method** | **Linear range (nmol/L)** | **Working curve** | **Coefficient** | **DL**  **(nmol/L)** |
| --- | --- | --- | --- | --- | --- |
| COF_TpBD-SS_ | SERS | 0.025-0.95 | ΔI_1615cm_^-1^=3015.2C+ 103.1 | 0.9862 | 0.012 |
|  | Abs | 0.05-0.75 | ΔA_530nm_= 0.274C+ 0.0033 | 0.9935 | 0.041 |

**TABLE S4. Comparison of analysis characteristics between this method and the reported method of Cd^2+^**

| **Method^*^** | **Method principle** | **Linear range**  **(nmol/L)** | **DL (nmol/L)** | **Annotation** | **Ref.** |
| --- | --- | --- | --- | --- | --- |
| FL | Cd^2+^ bound to PT probes to enhance fluorescence. | 18 - 300 | 14.5 | High sensitivity, good selectivity, but complicated operation and high cost. | (34) |
| ECL | Cd^2+^ bound to glutathione (GSH) PT in the detection electrode to cause signal changes. | 100 - 1500 | 63 | Fast and convenient, high accuracy, but low detection sensitivity. | (35) |
| FL | PT fluorescence chemical sensor DSC (Dan-Ser-Cys-NH_2_) based on photoinduced electron transfer (PET) detection Cd^2+^. | 20 -200 | 13.8 | It is fast and convenient, but the preparation process is complex and the cost is high. | (5) |
| CM | A highly sensitive colorimetric sensor strip (CCSS) based on cellulose film was prepared, and a sensitive and efficient visual colorimetric detection of Cd^2+^ was established. | 0.05-20 (mg/L) | 0.01 (mg/L) | Simple operation, low cost, but low sensitivity and accuracy. | (36) |
| SERS | The PT combined with Cd^2+^ to form a complex, detached from the surface of COF_TpBD-SS_, and restored its catalysis, which enhanced the SERS signal. | 0.025 – 0.95 | 0.012 | Sensitive, simple, fast, but high cost. | This assay |

* FL-fluorescence, CM- colorimetry, ECL-electrochemiluminescence.

**TABLE S5. Influence of interfering ions on SERS determination of Cd^2+^**

| **Interfering ion** | **Relative multiple** | **Relative error (%)** | **Interfering ion** | **Relative multiple** | **Relative error (%)** |
| --- | --- | --- | --- | --- | --- |
| Mg^2+^ | 1000 | 3.4 | Ca^2+^ | 1000 | -7.6 |
| Fe^3+^ | 1000 | 1.8 | Zn^2+^ | 1000 | 3.5 |
| K^+^ | 1000 | 5.4 | Al^3+^ | 1000 | -4.5 |
| Na^+^ | 1000 | -2.7 | Hg^2+^ | 100 | -3.3 |
| Ba^2+^ | 1000 | -3.8 | NH_4_^+^ | 100 | 6.8 |
| Co^2+^ | 1000 | 0.8 | Mn^2+^ | 1000 | 0.4 |
| Cr^6+^ | 100 | 7.0 | Cu^2+^ | 1000 | -3.4 |
| NO_2_^-^ | 100 | -3.9 | PO_4_^3-^ | 10 | 6.8 |
| Br^--^ | 10 | -4.7 | CO_3_^2-^ | 10 | -0.6 |
| I^-^ | 100 | 9.6 | SO_4_^2-^ | 100 | 7.5 |

**TABLE S6. SERS measurement results of** **Cd^2+^ in the samples**

| **Sample** | **Average（nmol/L）** | **Added Cd^2+^（nmol/L）** | **Found（nmol/L）** | | **Recovery**  **(%)** | **RSD（%）** | **Content** |
| --- | --- | --- | --- | --- | --- | --- | --- |
| Hubei rice | 0.2935 | 0.075 | 0.3115 | 102.4 | | 6.9 | 7.547 ng/g |
| Hunan rice | 0.3798 | 0.075 | 0.3871 | 95.3 | | 9.7 | 12.21 ng/g |
| Guangxi rice | 0.2733 | 0.075 | 0.2953 | 91.2 | | 6.8 | 6.733 ng/g |
| Wuchang rice | 0.1005 | 0.075 | - | 107.4 | | 1.9 | - |
